# Repeated misdiagnosis of squamous cell carcinoma of the thyroid: a case report

**DOI:** 10.3389/fonc.2025.1551514

**Published:** 2025-02-18

**Authors:** Zheng Yu, Ding Tingting, Zhang Jianyong, Peng Yunsong

**Affiliations:** ^1^ Breast Disease Center, The Affiliated Hospital of Guizhou Medical University, Guiyang, Guizhou, China; ^2^ Department of Pathology, The Affiliated Hospital of Guizhou Medical University, Guiyang, Guizhou, China; ^3^ Department of Vascular and Thyroid Surgery, Guizhou Provincial People’s Hospital, Guiyang, Guizhou, China; ^4^ Department of Imaging, Guizhou Provincial People’s Hospital, Guiyang, Guizhou, China

**Keywords:** squamous cell carcinoma of thyroid, thyroid tumor, subacute thyroiditis, misdiagnosis, prognosis

## Abstract

Squamous cell carcinoma of the thyroid (SCCT) represents a rare subtype of thyroid malignancy characterized by a poor prognosis and is frequently misdiagnosed as subacute thyroiditis (SAT). This report discusses a case of SCCT that was recurrently misdiagnosed with neck pain as the initial clinical presentation. An 80-year-old Asian female presented multiple times to the department of emergency, department of vascular and thyroid surgery, and department of endocrinology due to a painful neck mass. Ultrasonography identified a cystic solid mass in the right thyroid lobe, measuring 4.55 cm*3.2 cm. Enhanced computed tomography (CT) scanning revealed a significant enlargement of the right thyroid lobe, with an irregular mass exhibiting indistinct boundaries and blurred surrounding spaces. Laryngoscopic examination demonstrated immobilization of the right vocal cord and inadequate glottic closure. The erythrocyte sedimentation rate (ESR) was elevated to 94 mm/h. Thyroid function tests indicated subclinical thyrotoxicosis. The patient was initially diagnosed with subacute thyroiditis, and her symptoms improved following treatment with glucocorticoids and analgesics. One month following treatment, the patient experienced dyspnea, and subsequent computed tomography (CT) imaging revealed tracheal compression and narrowing, which contributed to the respiratory distress. A biopsy of the tumor was conducted, and pathological examination confirmed the presence of squamous cell carcinoma. To definitively diagnose SCCT, pathological examination and immunohistochemical analysis are required. Early and accurate diagnosis is essential for developing appropriate treatment strategies and enhancing patient survival rates. It is imperative for clinicians to comprehend this rare and life-threatening disease to enhance treatment efficacy.

## Introduction

Squamous cell carcinoma of the thyroid (SCCT) represents a rare subtype of thyroid malignancy, comprising approximately 0.1-0.4% of all thyroid cancer cases (1–3). This neoplasm is characterized by its highly aggressive nature and poor prognosis. Survival rates are notably low and the median survival duration ranges from 7.7 to 9.1 months (2–4). Patients with SCCT typically present at an advanced stage due to the prevalence of local metastases (55.4%), extrathyroidal extension (64%), and distant metastases (11.7%) (2, 4). The clinical manifestations of SCCT are predominantly influenced by tumor size and anatomical location, manifesting as a neck mass, hoarseness due to recurrent laryngeal nerve invasion, dyspnea from tracheal compression, and dysphagia resulting from esophageal invasion (3, 5). Previous findings suggest that laryngoscopy of vocal cord function is effective in evaluating the recurrent laryngeal nerve function ahead of surgery (6). In contrast, subacute thyroiditis (SAT) is an inflammatory disorder of the thyroid gland, the pathogenesis and clinical course determinants of which have remained elusive for decades (7). Women constitute 75-80% of SAT cases (7, 8). The predominant symptoms reported by SAT patients include anterior neck pain, frequently radiating to the jaw, ears, and upper mediastinum, as well as nocturnal fever (7–9). The most common laboratory finding in SAT is an elevated erythrocyte sedimentation rate (ESR), which can sometimes reach values in the triple digits (7, 9). C-reactive protein (CRP) levels are also elevated in many cases, although CRP is considered a less specific marker for SAT (9). Additionally, there may be an increase in the white blood cell count (WBC) (7, 9). The treatment regimen for SAT typically involves the administration of non-steroidal anti-inflammatory drugs (NSAIDs) to alleviate pain and inflammation, along with corticosteroids to further reduce inflammation (10). Subacute thyroiditis is generally self-limiting, with most patients recovering spontaneously within a few weeks to several months, necessitating regular follow-up (7). In this report, we present a case of SCCT that was repeatedly misdiagnosed with neck pain as the initial clinical manifestation.

## Case presentation

**Figure 1:**
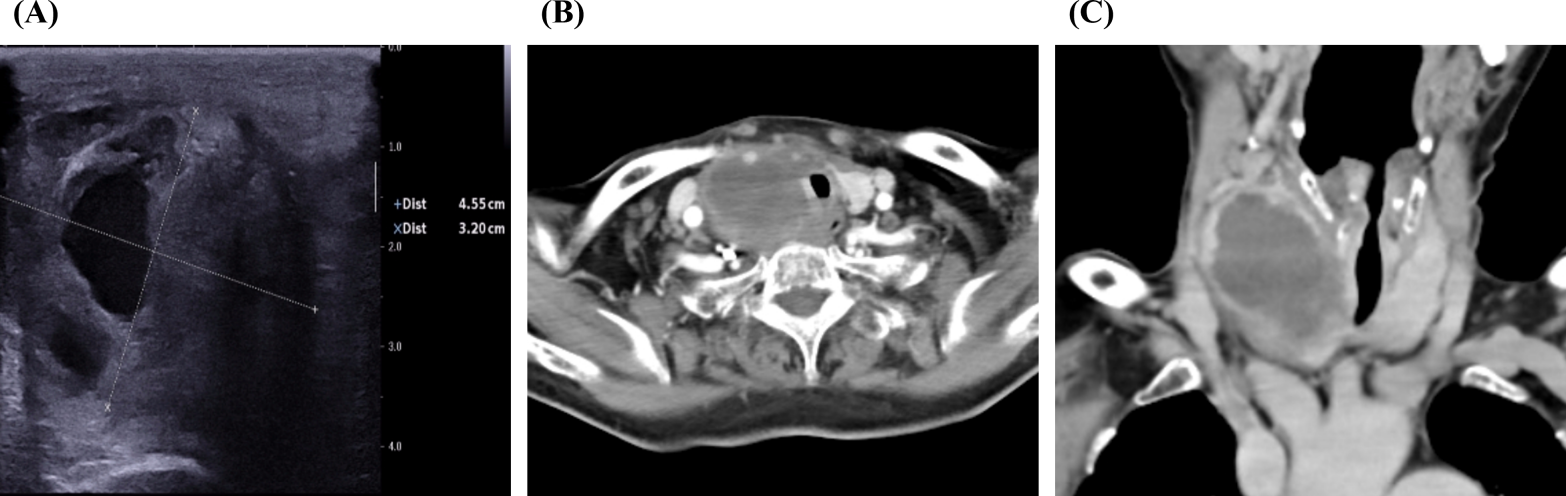
Typical image of imaging examination. **(A)** Ultrasound image of the tumor. **(B)** CT image of thyroid tumor in coronal section. **(C)** CT image of thyroid tumor in axial section.

**Figure 2:**
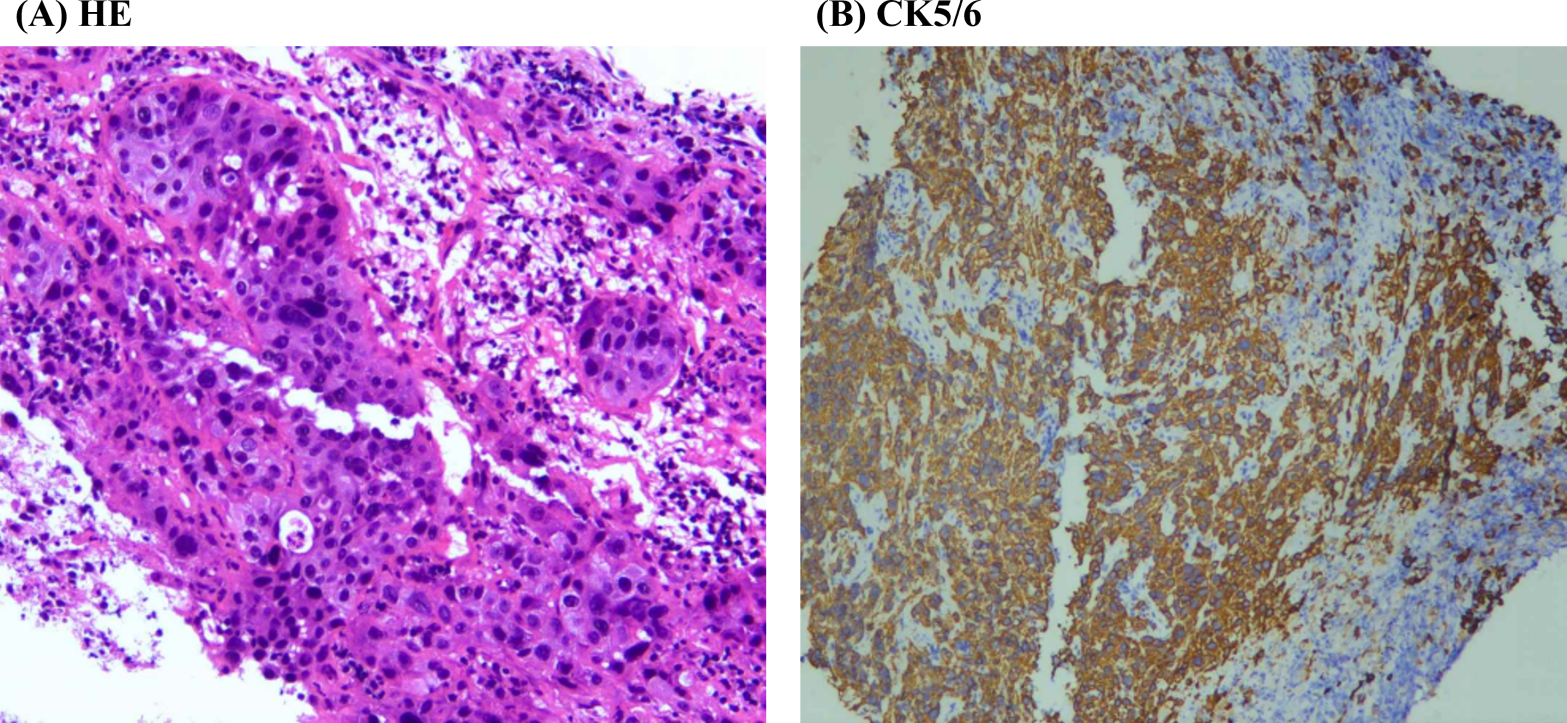
Typical diagram of pathological examination. **(A)** HE staining image of thyroid tumor. **(B)** Immunohistochemical results of CK5/6 in the thyroid tumor.

The patient, an 80-year-old Asian woman with no history of smoking, presented multiple times to the department of emergency, the department of vascular and thyroid surgery, and the department of endocrinology over the past three months due to a painful neck mass. Ultrasound examination identified a cystic solid mass in the right thyroid lobe measuring 4.55 cm*3.2 cm (**Figure 1A**). The tumor exhibited an indistinct boundary, a regular shape, a predominance of solid components, and an absence of blood flow signals. An enhanced computed tomography (CT) scan revealed a significant increase in the volume of the right thyroid lobe, characterized by an irregular mass with indistinct boundaries and blurred surrounding spaces. The adjacent structures were compressed and displaced, and the trachea was compressed and shifted to the left. The lesion measured approximately 50mm*42mm*60mm and exhibited heterogeneous enhancement post-contrast, with patchy areas of non-enhancing necrosis observed internally (**Figures 1B, C**). Laryngoscopic examination indicated immobilization of the right vocal cord and inadequate glottic closure. The ESR was elevated to 94 mm/h. Laboratory tests showed increased levels of free thyroxine 4, thyroglobulin antibodies, thyroperoxidase antibodies, and thyroglobulin, while thyrotropin-stimulating hormone levels were decreased. Based on these findings, the patient was diagnosed with subacute thyroiditis, and her symptoms improved following treatment with glucocorticoids and analgesics. However, one-month post-treatment, the patient developed dyspnea, and subsequent CT imaging demonstrated tracheal compression and narrowing, contributing to the respiratory distress. A biopsy of the tumor was performed, revealing squamous cell carcinoma upon pathological examination. Immunohistochemical analysis showed positive staining for CKpan, CK5/6 (**Figure 2**), P40, P63, and CK7, while negative staining was observed for NapsinA, TTF-1, CD56, CgA, Syn, Pax-8, Tg, and Vimentin. The Ki67 proliferation index was determined to be 70%. These results confirmed the diagnosis of SCCT. Suffering from shortness of breath, the patient was treated with a tracheal stent and later moved to the oncology department for further care. Three months after being diagnosed, the patient is still alive.

## Discussion

SCCT is an exceedingly rare and highly aggressive malignancy, typically associated with a poor prognosis and a survival rate often less than one year (3). The literature frequently recommends ultrasonography, computed tomography (CT)/magnetic resonance imaging (MRI), and fine needle aspiration cytology (FNAC) as diagnostic tools for thyroid nodules (11). Despite the availability of various diagnostic methods, including clinical assessment, diagnostic imaging, and endoscopy, early diagnosis of SCCT remains challenging (12). Particularly when the clinical presentation involves a sudden painful neck mass, there is a high risk of misdiagnosis as SAT. Furthermore, treatment for SAT often alleviates patient pain, which can further contribute to diagnostic errors. In our study, the patient was initially diagnosed with SAT; however, the definitive diagnosis was not established until she was readmitted to the hospital due to dyspnea and subsequently underwent a pathological examination.

SCCT typically occurs in an older demographic compared to conventional papillary thyroid carcinoma, with 76% of cases diagnosed in individuals aged between 60 and 80 years, and over 60% of reported cases occurring in those aged 60 or older (3, 13, 14). Patients are commonly present with a rapidly enlarging neck mass that invades adjacent structures and is often associated with cervical lymphadenopathy. The mass effect of the tumor can lead to obstructive symptoms, including dysphagia, dyspnea, hoarseness, and neck pain (14). The prognosis for patients with thyroid squamous cell carcinoma is generally poor, with a documented median survival of 8 months and a 2-year survival rate of 14% (3, 14). SCCT requires aggressive treatment involving a multidisciplinary approach. Patients who undergo radical surgery exhibit the highest survival rates (15, 16), while the response to radiotherapy is poor, and the tumor is relatively resistant to chemotherapy (1, 3, 4, 13, 17). At present, surgery is seen as an effective way to lessen tumor load and symptoms of local invasion, with successful surgical removal of SCCT tumors linked to better median overall survival (2), but radical surgery might lead to more severe complications. Additionally, SCCT does not uptake iodine, rendering radioactive iodine therapy ineffective (3). SCCT is an aggressive malignant neoplasm characterized by low incidence and poor prognosis (18–20). Prompt and precise diagnosis is crucial for formulating effective treatment strategies and enhancing patient survival rates. In the present case, a lack of clinical experience resulted in a misdiagnosis of SAT for a duration of three months, thereby overlooking the presence of SCCT.

Definitive diagnosis of thyroid cancer relies on pathological examination and immunohistochemical analysis. In primary thyroid squamous cell carcinomas, thyroglobulin and thyroid transcription factor-1 (TTF-1) are infrequently detected (21, 22), with previous studies indicating positivity rates of only 4% (1/25) and 17% (3/18), respectively (3). In thyroid malignancies, excluding well-differentiated thyroid cancers, the paired box gene 8 (PAX-8) protein is considered a more sensitive indicator of thyroid origin than TTF-1 or thyroglobulin (3); however, PAX-8 is present in limited quantities in primary squamous cell carcinomas. The proteins p63 and p40 serve as sensitive markers for squamous cell differentiation, with p40 being more specific than p63 in distinguishing squamous cell carcinoma (23). Notably, all 10 cases of primary thyroid squamous cell carcinoma reported in the literature demonstrated p63 positivity (3). Cytokeratin 5/6, a high molecular weight cytokeratin, serves as a marker for squamous cell carcinoma (24). In the literature, cytokeratin 5/6 has consistently tested positive for primary thyroid squamous cell carcinoma. Additionally, cytokeratin 7 was positive in 13 out of 14 reported cases of primary thyroid squamous cell carcinoma (3). In our case, the tumor exhibited positive staining for CK5/6, P40, P63, and CK7, while showing negative staining for Napsin A, TTF-1, CD56, Chromogranin A, Synaptophysin, Pax-8, thyroglobulin, and vimentin. These findings confirmed the diagnosis of SCCT.

The patient in our case report was repeatedly misdiagnosed with SAT due to several factors. Firstly, the patient presented with a sudden painful thyroid tumor and elevated ESR, making it challenging to differentiate from SAT. Secondly, SCCT is exceedingly rare and scarcely documented in the literature, resulting in a lack of awareness among some medical professionals. Through this case report, we aim to enhance clinicians’ understanding of SCCT and emphasize the importance of distinguishing it from SAT. Early and accurate diagnosis is crucial for formulating appropriate treatment strategies and improving patient survival rates. It is important for clinicians to understand this uncommon and life-threatening disease to improve treatment effectiveness.

## Additional Contributions

We thank the patient for granting permission to publish this information.

## Data Availability

The raw data supporting the conclusions of this article will be made available by the authors, without undue reservation.
